# Comparative analysis of cryoballoon versus radiofrequency catheter ablation in atrial fibrillation patients with impaired left ventricular ejection fraction

**DOI:** 10.1016/j.ijcha.2025.101721

**Published:** 2025-06-19

**Authors:** J. Pongratz, K.-H. Kuck, A. Metzner, U. Hink, A. Garcia Alberola, R. Borchard, G. Nölker, M. Kuniss, R.R. Tilz, J.W. Schrickel, A. Thornton, D. Thomas, H.A. Katus, R. Zahn, S. Spitzer, J.J. Souza, J. Brachmann, J. Tebbenjohanns, L.-Q. Wu, W.P. Obel, G. Groschup, C. Stellbrink, K.R.J. Chun, J.-H. Gerds-Li, A. Stanley, R.R. Gopal, L. Lickfett, A. Lubinski, B. Schumacher, C. Steinwender, H.A. Franke, T. Ouarrak, U. Dorwarth, J. Senges, E. Hoffmann, F. Straube, for the FREEZE Cohort Study Investigators

**Affiliations:** a Heart Center Munich Bogenhausen, Cardiology and Internal Intensive Care Medicine, Munich Municipal Hospital Group, Munich, Germany; b Klinik für Rhythmologie, Universitätsklinikum Schleswig-Holstein, Lübeck, Germany; c Klinik für Kardiologie / Elektrophysiologie, Universitäres Herz- und Gefäßzentrum Hamburg GmbH, Hamburg, DE, Germany; d Klinik für Innere Medizin 1 - Kardiologie, Klinikum Frankfurt Höchst GmbH, Frankfurt am Main, DE, Germany; e Department of Cardiology, University Hospital Virgen de la Arrixaca, El-Palmar, Murcia, Spain; f Contilia Herz- und Gefäßzentrum, Klinik für Kardiologie und Angiologie, Essen, Germany; g Internal Medicine II / Cardiology, Christian Hospital Unna-Mitte, Unna, Germany; h Department of Cardiology, Kerckhoff-Klinik, Bad Nauheim, Germany; i Medizinische Klinik und Poliklinik II, Universitätsklinikum Bonn, Bonn, Germany; j Center for Electrophysiology, Bremen, Germany; k Sunninghill Hospital, Johannesburg, South Africa; l Klinik für Innere Med. III, Kardiologie, Angiologie u. Pneumologie, Universitätsklinikum Heidelberg, Heidelberg, Germany; m Heidelberger Zentrum für Herzrhythmusstörungen (HCR), Medizinische Universitätsklinik Heidelberg, Heidelberg, Germany; n Klinikum Ludwigshafen, Ludwigshafen am Rhein, Germany; o Praxis Klinik Herz und Gefäße, Dresden, Germany; p Department of Cardiology, Mission Hospital, Asheville, United States; q Medical School / Regiomed GmbH, Coburg, Germany; r Med. Klinik I, Helios Klinikum Hildesheim GmbH, Hildesheim, Germany; s Dept. Of Cardiology, Shanghai Rui Jin Hospital, Shanghai, PR China; t Department of Cardiology, Milpark Hospital, Parktown West, South Africa; u Medizinische Klinik IV, Klinikum Hanau GmbH, Hanau, Germany; v Klinik für Kardiologie und intern. Intensivmedizin, Klinikum Bielefeld Mitte, Bielefeld, Germany; w Medizinische Klinik III – CCB, Agaplesion Markus Krankenhaus, Frankfurt am Main, Germany; x Deutsches Herzzentrum der Charite, Berlin, Germany; y Cardiology, Netcare Sunninghill Hospital, Sandton, South Africa; z Cardiology, Panorama Medi-Clinic, Cape Town, South Africa; aa Gemeinschaftspraxis Kardiologie und Pneumologie, Mönchengladbach, Germany; ab Department of Cardiology and Internal Diseases, Medical University of Gdańsk, Gdańsk, Poland; ac Klinik für Innere Medizin II, Westpfalz-Klinikum GmbH, Kaiserslautern, Germany; ad Kardiologie und internistische Intensivmedizin, Kepler Universitätsklinikum, Linz, Austria; ae Abteilung Kardiologie, Klinikum Siloah, Hannover, Germany; af Stiftung Institut für Herzinfarktforschung, Ludwigshafen, Germany

**Keywords:** Atrial fibrillation, Cryoballoon ablation, Radiofrequency ablation, Catheter ablation, Impaired ejection fraction, recurrence, safety

## Abstract

**Background:**

In selected patients with atrial fibrillation (AF) and impaired left ventricular ejection fraction (LVEF), catheter ablation has been proposed for rhythm control. It is unclear, if cryoballoon (CBA) or radiofrequency (RFA) ablation is the preferred technique.

**Methods:**

The FREEZE Cohort (NCT01360008) sub-analysis included patients with LVEF < 50 % undergoing CBA (Group A) or RFA (Group B) comparing baseline characteristics, procedural data and outcome.

**Results:**

From 2011 to 2016; 4,189 patients were enrolled, with 256 (6.1 %) qualifying for the sub-analysis and divided into two groups: 118 (Group A) and 138 (Group B). Mean age was 63.4 ± 9.9 years (p = 0.07), with 60.9 % suffering from persistent AF (p = 0.46). Mean LVEF was 42.0 % (p = 0.81). CHA_2_DS_2_-VASc Score was lower in Group A (p < 0.01). Group A had shorter procedure and left atrial times (p < 0.001) but higher fluoroscopy times (p < 0.001) and dose area products (p < 0.01). Acute PVI was achieved in 96.4 % (p = 0.57). Complications were lower in Group A (5.1 % vs. 13.1 %, p < 0.05). After 449 and 516 days (p < 0.001), no differences in arrhythmia recurrence were observed (51.2 % vs. 58.7 %, p = 0.30), but rehospitalizations were more frequent in Group B (34.9 % vs. 52.1 %, p < 0.05). A trend for more re-ablations was observed in Group B (11.5 vs. 22.1 %, p = 0.06). Female sex was the sole independent predictor of arrhythmia recurrence.

**Conclusion:**

CBA procedures were associated with lower rates of complications, fewer rehospitalizations, and shorter procedural times, whereas RFA procedures resulted in lower radiation exposure. Overall, AF ablation in patients with impaired LVEF is an effective initial ablation strategy with either RFA or CBA.

## Graphical abstract

**Figure:**
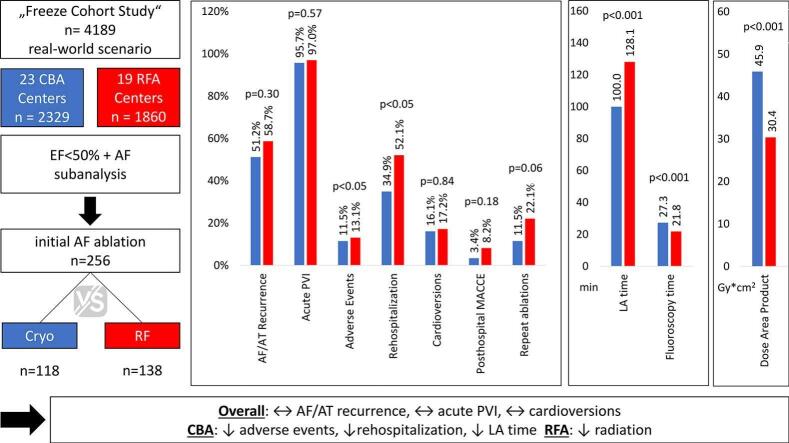

## 1 Introduction

In symptomatic atrial fibrillation (AF) patients pulmonary vein isolation (PVI) by means of catheter ablation (CA) is a well-established treatment option [1]. However, the outcome of the procedure is greatly affected by the individual patient's morbidity, as underlying conditions contribute to the inception, perpetuation, and advancement of AF [[1], [2], [3]]. The management of patients with AF and coincident heart failure (HF) with a reduced left ventricular ejection fraction (LVEF) poses a unique challenge due to altered hemodynamics, leading to significant structural alterations and setting off a perpetuating cycle of deterioration [1,4]. In addition to optimal pharmacological therapy for HF, evidence demonstrates significant benefits of CA for these patients: The CASTLE-AF trial demonstrated a reduction of all-cause mortality and hospitalization for worsening HF after AF ablation in patients with reduced LVEF [5]. According to PABA-CHF study, AF CA has been shown to improve symptoms, exercise capacity, quality of life, and LVEF in HF [6]. Recently, randomized data has shown that among patients with AF and end-stage HF, the combination of CA and guideline-directed medical therapy was associated with a lower likelihood of a composite of death from any cause, implantation of a left ventricular assist device, or urgent heart transplantation than medical therapy alone [7]. In addition, recent observational data support the safety and efficacy of AF ablation in patients with LVEF < 50 %, including those with mid-range ejection fraction. Sakamoto et al. reported significant improvements in cardiac and renal function, low complication rates, and freedom from recurrence in a mixed cohort of patients with mildly to moderately reduced LVEF undergoing AF ablation [8].

In general, the recommended ablation strategy is pulmonary vein electrical isolation, and radiofrequency ablation (RFA) or cryoballoon ablation (CBA) are established interventional techniques [9]. RFA is a point-by-point technique offering greater flexibility, potentially suitable for treating atrial arrhythmias beyond the pulmonary veins (PV). In contrast, CBA is designed as a single-shot tool, specifically targeting the PV ostia to achieve electrical en-bloc isolation. A randomized trial in symptomatic paroxysmal AF comparing both techniques demonstrated that CBA was noninferior to RFA in terms of efficacy without differences in safety. Real-world data in paroxysmal or persistent AF demonstrated lower rates of rehospitalization due to re-ablations and adverse events during follow-up after CBA than after RFA [10].

The EAST-AFNET 4 randomized controlled trial recently demonstrated that early rhythm control treatment lowers the risk of adverse cardiovascular outcomes [11]. In a treatment-related outcome analysis of the AF patients with HF enrolled in the CABANA trial (Catheter Ablation Versus Antiarrhythmic Drug Therapy for Atrial Fibrillation), patients with AF who had clinically diagnosed stable HF at trial entry, CA produced clinically important improvements in survival, freedom from AF recurrence, and quality of life relative to drug therapy [12].

It remains unclear whether patients with AF and HF are better treated with RFA or CBA in terms of efficacy and safety in the initial ablation procedure. Most studies on CA in AF with LV dysfunction have focused on RFA, with fewer studies investigating CBA. The CBA technique has potential advantages and disadvantages in HF patients with AF. Advantages may include shorter procedural time, reduced complexity, and a lower rate of repeat ablations. However, a disadvantage could be that secondary arrhythmias outside the PV are not addressable with the cryoballoon, which are more common in sicker patients [13].

Since there is no randomized trial data evaluating the efficacy and safety of CBA and RFA in AF patients with non-preserved LVEF, the primary objective of this sub-analysis study was to investigate differences in CBA and RFA procedures in HF patients with impaired LVEF regarding outcomes, procedural results, safety, and clinical course. The secondary objective was to analyze predictors of arrhythmia recurrence for this specific group of patients.

## 2 Methods

### 2.1 Study design

The results from the primary endpoint analysis of the FREEZE Cohort Trial (ClinicalTrials.gov Identifier: NCT01360008), a prospective-cluster-cohort study, have been published and the methodologies have been detailed previously [14]. A general outline of the procedural approach for both CBA and RFA is provided in the supplementary Methods.

This sub-analysis specifically targeted patients with impaired LVEF (LVEF < 50 %) who underwent either CBA in group A or RFA in group B for PVI, leading to the formation of two distinct patient groups.

Comparative assessments of baseline characteristics, procedural data, and outcome data were conducted between the groups.

### 2.2 Statistical analysis

Categorical variables were presented as counts and percentages and were compared by chi-square test.

Continuous variables were presented as median and quartiles or mean and standard deviation and were compared by Mann-Whitney-Wilcoxon test. Predictors of atrial arrhythmia recurrence were analyzed using logistic regression analysis. Corresponding OR with 95 %-CI were presented. All tests were 2-tailed and p-values < 0.05 were considered statistically significant. All analyses were performed using SAS statistical software package, version 9.4 (Cary, NC, USA).

## 3 Results

### 3.1 Study population

**Fig. 1:**
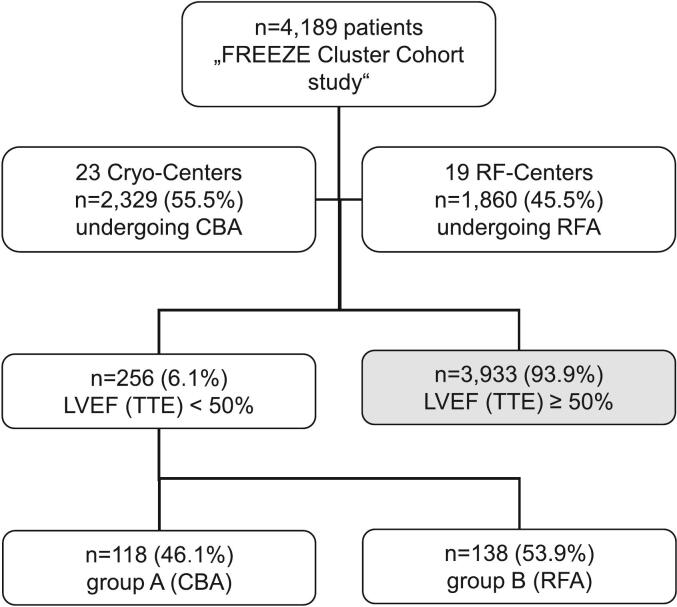
**Study population flow chart.** This flowchart illustrates the characterization of the current study population. The uppermost box represents the total number of patients encompassed in the FREEZE cluster cohort study. Each branch delineates a distinct stage of the selection process. CBA: cryoballoon ablation, RFA: radiofrequency ablation, TTE: transthoracic echocardiography.

Between 2011 and 2016, a total of 4,189 patients were enrolled in the FREEZE Cluster Cohort study, and this analysis specifically included 256 individuals (6.1 %) (see Fig. 1 for details). The exclusion criteria for the analysis were applied to the remaining 3,933 patients, as their EF was greater than or equal to 50 %.

The study population was stratified into two groups, as previously outlined [Group A: 118 patients undergoing CBA (46.0 %), Group B: 138 patients undergoing RFA (54.0 %)]. The collective mean age of the study participants was 63.4 ± 9.9 years (p = 0.07), with 26.6 % being female (p = 0.64), and 60.9 % experiencing persistent AF (p = 0.46). The mean LVEF was 42.0 % (p = 0.81), and the median left atrial (LA) diameter measured 47.0 mm (p = 0.60). There was no difference observed regarding pulmonary vein variants like left common ostium or accessory pulmonary veins in both groups (p = 0.84, p = 0.92). Patients with moderately and severely reduced LVEF were distributed equally (p = 0.55, p = 0.10).

In comparing the two groups, group A exhibited a lower percentage of severe or disabling EHRA symptom scores (44.9 % vs. 78.3 % and 3.4 % vs. 2.2 %, p < 0.0001), along with a reduced prevalence of valve disease ≥ grade 2 (8.5 % vs. 34.8 %, p < 0.0001), mitral regurgitation ≥ grade 2 (3.4 % vs. 21.7 %, p > 0.0001), chronic kidney disease (8.4 % vs. 17.4 %, p < 0.05), structural heart disease (67.8 % vs. 79.7 %, p < 0.05) and a lower CHA_2_DS_2_-VASc-Score (2.3 ± 1.4 vs. 2.8 ± 1.5, p < 0.01). The mean number of AADs used per patient (excluding beta-blockers) was 1.1 ± 0.7 overall (1.2 ± 0.9 vs. 1.0 ± 0.6, p = 0.05). Please refer to Table 1 for further details.

**Table 1:** Baseline characteristics and comparison of groups.

	**Total (n = 256)**	**Group A (n = 118)**	**Group B (n = 138)**	**p-value**
**Age, years**	63.4 ± 9.9	62.0 ± 10.1	64.6 ± 9.7	0.07
**Females (%)**	68 (26.6)	33 (28.0)	35 (25.4)	0.64
**Persistent AF (%)**	156 (60.9)	69 (58.5)	87 (63.0)	0.46
**Inefficacy of at least 1 AAD (%)**	236 (92.2)	110 (93.2)	126 (91.3)	0.15
**BMI, kg/m^2^**	28.2 ± 4.6 (n = 245)	28.1 ± 4.7 (n = 114)	28.4 ± 4.5 (n = 131)	0.51
**EHRA**				**< 0.0001**
**I/no (%)**	11 (4.3)	9 (7.6)	2 (1.4)	
**II/mild (%)**	77 (30.1)	52 (44.1)	25 (18.1)	
**III/severe (%)**	161 (62.9)	53 (44.9)	108 (78.3)	
**IV/disabling (%)**	7 (2.7)	4 (3.4)	3 (2.2)	
**Frequency of episodes**				0.20
**Occasionally (<1x/month)**	21/240 (8.8)	16/108 (14.8)	5/132 (3.8)	
**Intermediate (>1x/month)**	172/240 (71.7)	70/108 (64.8)	102/132 (77.3)	
**Frequent (daily)**	47/240 (19.6)	22/108 (20.4)	25/132 (18.9)	
**Previous device implantation (%)**	53 (20.7)	12 (10.2)	41 (29.7)	**< 0.001**
**Pacemaker (%)**	16/53 (30.2)	7/12 (58.3)	9/41 (22.0)	**< 0.01**
**ICD (%)**	28/53 (52.8)	4/12 (33.3)	24/41 (58.5)	0.12
**Other (%)**	9/53 (17.0)	1/12 (8.4)	8/41 (19.5)	0.18
**Coronary heart disease (%)**	59 (23.0)	22 (18.6)	37 (26.8)	0.12
**Valve disease ≥ grade 2 (%)**	58 (22.7)	10 (8.5)	48 (34.8)	**< 0.0001**
**Mitral regurgitation ≥°II (%)**	34 (13.3)	4 (3.4)	30 (21.7)	**< 0.0001**
LA diameter*, mm	47.0 (42.0, 52.5)	48.0 (42.0, 54.0)	47.0 (42.0, 52.0)	0.60
Ejection fraction* (%)	42.0 (35.0, 45.0)	42.5 (35.0, 45.0)	41.5 (35.0, 45.0)	0.81
**Moderately reduced EF^†^ (%)**	82 (32.0)	40 (33.9)	42 (30.4)	0.55
**Severely reduced EF^‡^ (%)**	36 (14.1)	12 (10.2)	24 (17.4)	0.10
**CHA_2_DS_2_-VASc-Score**	2.6 ± 1.4	2.3 ± 1.4	2.8 ± 1.5	**< 0.01**
**Hypertension (%)**	180 (70.3)	76 (64.4)	104 (75.4)	0.06
**CKD (%)**	34 (13.3)	10 (8.5)	24 (17.4)	**< 0.05**
**Diabetes mellitus (%)**	30 (11.7)	10 (8.5)	20 (14.5)	0.14
Hypertensive heart disease* (%)	56 (21.9)	32 (27.1)	24 (17.4)	0.06
**Cardiomyopathy (%)**	81 (31.6)	35 (29.7)	46 (33.3)	0.53
**DCM (%)**	52/81 (64.2)	21/35 (60.0)	31/46 (67.4)	0.49
**HCM (%)**	3/81 (3.7)	2/35 (5.7)	1/46 (2.2)	0.40
**Other (%)**	29/81 (35.8)	12/35 (34.3)	17/46 (37.0)	0.80
**Prior stroke (%)**	14 (5.5)	6 (5.1)	8 (5.8)	0.80
**Structural heart disease (%)**	190 (74.2)	80 (67.8)	110 (79.7)	**< 0.05**
**AAD medication**	1.1 ± 0.7	1.2 ± 0.9	1.0 ± 0.6	**0.05**
**Left common ostium (%)**	16/156 (10.3)	10/101 (9.9)	6/55 (10.9)	0.84
**Accessory veins (%)**	6/156 (3.8)	4/101 (4)	2/55 (3.6)	0.92

n (%), Mean ± SD, or Median (Quartiles) as appropriate according to the test of normal distribution. AF: atrial fibrillation, AAD: antiarrhythmic drug, DCM: dilated cardiomyopathy, HCM: hypertrophic cardiomyopathy, EHRA: European heart rhythm association, CKD: chronic kidney disease, BMI: body mass index, ICD: implantable cardioverter-defibrillator, LA: left atrium, EF: ejection fraction. *determined by transthoracic echocardiography. †: defined as LVEF 31 % − 40 %. ‡: defined as LVEF ≤ 30 %.

### 3.2 Procedural characteristics

The median procedure time [135.0 (99.0, 171.0) vs. 162.0 (122.0, 190.0) minutes), left atrial time [90.0 (70.0, 124.0) vs. 125.0 (90.0, 150.0) minutes), and total duration of all applications (36.1 ± 15.8 vs. 48.7 ± 22.9 min) were significantly shorter in group A compared to group B, respectively (all p < 0.001). The median dose area product [2878.0 (1426.0; 5295.0) vs. 2097.0 (1014.0; 3617.0) cGyxcm2, p < 0.01)] and mean fluoroscopy time (27.3 ± 14.6 vs. 21.8 ± 14.1 min, p < 0.001) were significantly higher in group A. Additional linear lesions were carried out more frequently in group B (11.2 % vs. 22.2 %, p < 0.05), with a significantly higher number of right atrial lines performed (0.0 % vs. 40.0 %, p < 0.01). PV anatomy was identified significantly more often in group A compared to group B (86.3 % vs. 39.9 %, p < 0.0001), while also showing a higher rate of intracardiac echocardiography usage (55.2 % vs. 4.4 %, p < 0.0001). PV angiography was performed at a similar frequency in both groups (77.6 % vs. 71.1 %, p = 0.24). Acute PVI could be achieved in 96.4 % of all patients (p = 0.57), group A exhibited a higher degree of procedural difficulty according to the treating physician (p < 0.001). The amount of both total (2.8 ± 0.9 vs. 3.4 ± 0.6, p < 0.0001) and LA catheters (1.1 ± 0.3 vs. 2.0 ± 0.2, p < 0.0001) used was significantly higher in group B. Procedural data is provided in Table 2 for further insight. At discharge, 8.2 % of patients received Class I AADs and 2.4 % Class III AADs, with similar distributions between groups (p = 0.21, p = 0.07).

**Table 2:** Procedural results.

	**Total (n = 256)**	**Group A (n = 118)**	**Group B (n = 138)**	**p-value**
**Total catheters (%)**	3.1 ± 0.8 (n = 251)	2.8 ± 0.9 (n = 116)	3.4 ± 0.6 (n = 135)	**< 0.0001**
**LA catheters (%)**	1.6 ± 0.5 (n = 250)	1.1 ± 0.3 (n = 116)	2.0 ± 0.2 (n = 134)	**< 0.0001**
**Electrophysiological Mapping (%)**	250/256 (97.7)	115/118 (97.5)	135/138 (97.8)	0.85
**Conventional (%)**	112/250 (44.8)	108/115 (93.9)	4/135 (3.0)	**< 0.0001**
**3D Mapping (%)**	138/250 (55.2)	7/115 (6.1)	131/135 (97.0)	**< 0.0001**
**Fluoroscopy Time, min**	24.4 ± 14.6 (n = 248)	27.3 ± 14.6 (n = 116)	21.8 ± 14.1 (n = 132)	**< 0.001**
Dose area Procuct, cGy*cm^2^	2397.0 (1193.0, 4764.0) (n = 245)	2878.0 (1426.0, 5295.0) (n = 113)	2097.0 (1014.0, 3617.0) (n = 132)	**< 0.01**
**LA Time, min**	110.0 (77.0, 141.0) (n = 243)	90.0 (70.0, 124.0) (n = 113)	125.0 (90.0, 150.0) (n = 130)	**< 0.0001**
**Procedure Time, min**	150.0 (110.0, 180.0) (n = 246)	135.0 (99.0, 171.0) (n = 115)	162.0 (122.0, 190.0) (n = 131)	**< 0.001**
**ICE usage, %**	70/251 (27.9)	64/116 (55.2)	6/135 (4.4)	**<0.0001**
**PV angiography, %**	186/251 (74.1)	90/116 (77.6)	96/135 (71.1)	0.24
**Additional linear lesions (%)**	43/251 (17.1)	13/116 (11.2)	30/135 (22.2)	**< 0.05**
**Left atrial (%)**	11/43 (25.6)	3/13 (23.1)	8/30 (26.7)	0.80
**Right atrial (%)**	12/43 (27.9)	0/13 (0.0)	12/30 (40.0)	**<0.01**
**LA isthmus (%)**	15/43 (34.9)	2/13 (15.4)	13/30 (43.3)	0.08
**Roof line LA (%)**	15/43 (34.9)	5/13 (38.5)	10/30 (33.3)	0.75
**RA isthmus (%)**	28/43 (65.1)	6/13 (46.2)	22/30 (73.3)	0.09
**Other (%)**	6/43 (14.0)	1/13 (7.7)	5/30 (16.7)	0.44
**Isolation of all PV (%)**	242/251 (96.4)	111/116 (95.7)	131/135 (97.0)	0.57
**LSPV (%)**	249/251 (99.2)	114/116 (98.3)	135/135 (100.0)	0.13
**RSPV (%)**	249/251 (99.2)	115/116 (99.1)	134/135 (99.3)	0.91
**LIPV (%)**	247/251 (98.4)	113/116 (97.4)	134/135 (99.3)	0.24
**RIPV (%)**	247/251 (98.4)	114/116 (98.3)	133/135 (98.5)	0.88
**Accessory (%)**	5/9 (55.6)	4/8 (50.0)	1/1 (100.0)	0.34
Degree of difficulty*				**< 0.001**
**Simple (%)**	146/250 (58.4)	55/116 (47.4)	91/134 (67.9)	
**Moderatetely aggravated (%)**	85/250 (34.0)	49/116 (42.2)	36/134 (26.9)	
**Severely aggravated (%)**	19/250 (7.6)	12/116 (10.3)	7/134 (5.2)	

n (%), Mean ± SD, or Median (Quartiles) as appropriate according to the test of normal distribution. LA: left atrium, PV: pulmonary vein, LSPV: left superior pulmonary vein, RSPV: right superior pulmonary vein, LIPV: left inferior pulmonary vein, RIPV: right inferior pulmonary vein. ICE: intracardiac echocardiography.

*The procedural degree of difficulty was rated by the operating electrophysiologist immediately after the ablation using a non-standardized, subjective scale based on perceived complexity, catheter handling, and anatomical or technical challenges.

### 3.3 Adverse events until hospital discharge

In the entire study population, total major adverse cerebrovascular and cardiac events (MACCE) included one stroke (0.4 %) among the group B. There were no deaths or myocardial infarctions documented.

Total procedural complications were less frequently observed in group A (5.1 % vs. 13.1 %, p < 0.05). Neither the in-hospital rates of MACCE nor major and minor complications, did exhibit significant differences between the two groups (p = 0.35, p = 0.24, p = 0.07). The sub-analysis of the groin complications treated conservatively revealed a significant difference in inguinal hematoma between group A and group B (0.0 % vs. 4.4 %, p < 0.05). For detailed information, please consult supplementary Table 1.

During the in-hospital period, AF recurrence until discharge was documented in 8.7 % of patients overall, with 3.4 % in the group A and 13.2 % in the group B (p < 0.01).

### 3.4 Outcome

Follow-up data were available for 95.3 % of patients (p = 0.14), with a median follow-up duration of 505 days (Group A: 449 days, Group B: 516 days, p < 0.001). No significant difference in arrhythmia recurrence was observed between the two groups (51.2 % vs. 58.7 %, p = 0.30). Subanalysis of patients with moderately reduced EF (42.9 % vs. 62.9 %, p = 0.11) and severely reduced EF (72.7 % vs. 64.3 %, p = 0.65) also showed no differences in recurrence rates.

Further subgroup analysis by AF type revealed no significant differences in arrhythmia recurrence for paroxysmal AF (46.2 % vs. 56.4 %, p = 0.36) and persistent AF (55.3 % vs. 60.0 %, p = 0.62).

Early arrhythmia recurrence within the first 3 months occurred in 33.3 % of patients (58/174), with no significant difference between groups (28.6 % vs. 37.1 %, p = 0.24).

Regarding the improvement of the EHRA symptom score, no statistically significant difference was noted (p = 0.74). At follow-up, 18.2 % were on Class III agents, predominantly amiodarone. Notably, amiodarone usage was significantly more frequent in group B compared to group A (100.0 % vs. 68.8 % of patients receiving Class III AADs, p < 0.05).

Among 31 patients who underwent repeat ablation (17.0 %), 23 procedures (74.2 %) were performed due to documented arrhythmia recurrence; however, no differentiation between AF and atrial tachycardia was available. In the remaining cases, the indication was either unrelated (n = 7, 22.6 %) or not clearly specified (n = 4, 12.9 %). There was a trend toward a higher rate of repeat ablations in group B compared to group A (22.1 % vs. 11.5 %, p = 0.06). Cardioversions were performed equally often in both groups (16.1 % vs. 17.2 %, p = 0.84). Group A exhibited a significantly lower percentage of rehospitalization after discharge (34.9 % vs. 52.1 %, p < 0.05). The reasons for rehospitalization showed no differences, whether rehospitalization was attributable to AF, other cardiovascular causes, or non-cardiovascular causes (p = 0.81).

Overall posthospital complication rates were similar in both groups (11.5 % vs. 15.3 %, p = 0.45). Neither posthospital MACCE, major or minor adverse events showed any statistical differences between the two groups (p = 0.18, p = 0.20, p = 0.88). Further details are provided in supplementary Table 2.

Subanalysis of patients with moderately (5.1 % vs. 14.6 %) and severely reduced EF (6.7 % vs. 4.0 %) showed similar adverse event rates for both ablation modalities (p = 0.16, p = 0.71).

Patients rated the procedure as “overall successful” in 48.2 % (p = 0.45) and “feeling safe during treatment” in 92.9 % (p = 0.75). The majority expressed a willingness to return to the same institution if necessary (94.1 %, p = 0.68). All data regarding the outcome is provided in Table 3.

**Table 3:** Follow-up data.

	**Total (n = 256)**	**Group A (n = 118)**	**Group B (n = 138)**	**p-value**
**Follow-up performed (%)**	244/256 (95.3)	110/118 (93.2)	134/138 (97.1)	0.14
**Days since intervention, days**	505.0 (419.0, 533.0)	449.0 (380.0, 527.0)	516.0 (444.0, 537.0)	**< 0.001**
**Arrhythmia recurrence (%)**	105/190 (55.3)	44/86 (51.2)	61/104 (58.7)	0.30
**AF recurrence until discharge (%)**	22/254 (8.7)	4/118 (3.7)	18/136 (13.2)	< **0.01**
**Early arrhythmia recurrence (%)**	58/174 (33.3)	22/77 (28.6)	36/97 (37.1)	0.24
**EHRA**				0.74
**I/no (%)**	127/170 (74.7)	61/83 (73.5)	66/87 (75.9)	
**II/mild (%)**	31/170 (18.2)	16/83 (19.3)	15/87 (17.2)	
**III/severe (%)**	12/170 (7.1)	6/83 (7.2)	6/87 (6.9)	
**IV/disabling (%)**	0/170 (0.0)	0/83 (0.0)	0/87 (0.0)	
**AAD class I medication (%)**	8/176 (4.5)	4/83 (4.8)	4/93 (4.3)	0.87
**AAD class III medication (%)**	32/176 (18.2)	16/83 (19.3)	16/93 (17.2)	0.72
**Amiodarone usage (%)**	27/32 (84.4)	11/16 (68.8)	16/16 (100)	< **0.05**
**Dronedarone usage (%)**	1/32 (3.1)	1/32 (6.3)	0/16 (0.0)	0.31
**Sotalol usage (%)**	3/32 (9.4)	3/16 (18.8)	0/16 (0.0)	0.07
**Other (%)**	1/32 (3.1)	1/32 (6.3)	0/16 (0.0)	0.31
**Repeat ablations (%)**	31/182 (17.0)	10/87 (11.5)	21/95 (22.1)	0.06
**Atrial fibrillation (%)**	23/31 (74.2)	6/10 (60.0)	17/21 (81.0)	0.21
**Other (%)**	7/31 (22.6)	4/10 (40.0)	3/21 (14.3)	0.11
**Unknown (%)**	4/31 (12.9)	1/10 (10.0)	3/21 (14.3)	0.74
**Cardioversions (%)**	30/180 (16.7)	14/87 (16.1)	16/93 (17.2)	0.84
**Rehospitalisation since discharge (%)**	79/180 (43.9)	30/86 (34.9)	49/94 (52.1)	**< 0.05**
**Causes of rehospitalization**				0.81
**Atrial fibrillation (%)**	37/78 (47.4)	14/30 (46.7)	23/48 (47.9)	
**Other CV cause (%)**	21/78 (26.9)	9/30 (30.0)	12/48 (25.0)	
**Non-CV cause (%)**	20/78 (25.6)	7/30 (23.3)	13/48 (27.1)	
**Patient satisfaction**				0.45
**Overall successful (%)**	95/197 (48.2)	41/79 (51.9)	54/118 (45.8)	
**Partially successful (%)**	34/197 (17.3)	14/79 (17.7)	20/118 (16.9)	
**Not successful (%)**	24/197 (12.2)	8/79 (10.1)	16/118 (13.6)	
**Not determinable (%)**	2/197 (1.0)	0/79 (0.0)	2/118 (1.7)	
**Unknown (%)**	41/197 (20.8)	15/79 (10.0)	26/118 (22.0)	
**Question not asked (%)**	1/197 (0.5)	1/79 (1.3)	0/118 (0.0)	
**Feeling safe (%)**	143/154 (92.9)	59/63 (93.7)	84/91 (92.3)	0.75
**Return to the same clinic (%)**	144/153 (94.1)	58/61 (95.1)	86/92 (93.5)	0.68

n (%), Mean ± SD, or Median (Quartiles) as appropriate according to the test of normal distribution. CV: cardiovascular. AAD: antiarrhythmic drug.

### 3.5 Evaluation of risk factors for arrhythmia recurrence

The independent risk factors for atrial arrhythmia recurrence, after a three-month blanking period, were determined using logistic regression analysis. The entered variables included RF energy utilization, LVEF in percentage, female sex, age on admission, paroxysmal atrial fibrillation, LA diameter in millimeters, and body mass index > 35. The analysis revealed that female sex was the only identified significant risk factor for atrial arrhythmia recurrence, showing an odds ratio of 2.53 and a p-value of 0.02. None of the other evaluated parameters demonstrated a significant impact on atrial arrhythmia recurrence in this multivariate model. Further details are available in supplementary Table 3.

## 4 Discussion

To the best of our knowledge, this is the most extensive dataset on AF patients with impaired LVEF currently available, focusing on the differences between two PVI techniques, CBA and RFA.

Furthermore, the uniqueness of these results is derived from the FREEZE Cohort Trial being a multicenter, multinational study that collected real-world data from centers adept in the specified ablation techniques. In this clustered cohort trial, experienced centers were pre-assigned to either RFA or CBA based on their preferred technique before the trial started [14].

The main findings of this sub-analysis are: Firstly, CBA and RFA are both effective and safe treatment strategies with equally high success rates in AF patients with impaired LVEF. Secondly, ablation procedures and LA times were shorter in the CBA, whereas fluoroscopy time and dose area product were higher. Thirdly, total procedural complications were less frequent in CBA, mainly due to the higher incidence of inguinal hematoma in RFA. Fourthly, CBA exhibited a significantly lower percentage of rehospitalization after discharge, while presenting equal rehospitalization causes and cardioversion frequencies. Fifthly, the female sex was identified as the sole independent risk factor for arrhythmia recurrence in AF patients with impaired LVEF.

The present study contributes to the growing body of evidence supporting the efficacy and safety of both CBA and RFA as treatment strategies for AF in patients with impaired LVEF. The findings demonstrate comparable success rates between the two approaches, emphasizing their effectiveness in achieving acute and durable PVI, while having a low rate for adverse events.

This equal effectiveness was also shown in the respective subanalysis for patients with moderately reduced LVEF as well as severely reduced LVEF, and for those with paroxysmal versus persistent AF.

Several studies have independently reported beneficial outcomes with RFA in AF patients with reduced LVEF. The CASTLE-AF trial investigated the outcome of catheter ablation compared to medical therapy in AF patients with a LVEF of 35 % or less, and an implanted defibrillator. The outcome demonstrated a freedom of atrial arrhythmia recurrence of about 60 % of the ablated patients after the first 12 months of follow up. Procedure related complications were documented in 7.8 % cases [5]. Similarly, in the PABA-CHF study, which compared RFA with atrioventricular node ablation plus biventricular pacing, freedom from AF after 6 months was 71 % in the RFA group. Adverse events were documented in 17.6 % patients [6].

In a study by Heeger et al., outcomes of CBA were specifically analyzed in patients with HF and reduced ejection fraction (LVEF ≤ 40 %). In this subgroup of 50 patients, 12-month arrhythmia-free survival (73.1 %) was comparable to that of matched patients with preserved LVEF, and improvements in NYHA class and LVEF were observed [15]. The major procedural complication rate was 8 %.

The parallel effectiveness observed in our study regarding RFA and CBA aligns with these established findings. In the current trial, the recurrence rates of arrhythmia were higher, which could be attributed to a longer follow-up period or potential differences in patient populations. Interestingly, in-hospital arrhythmia relapses were significantly less frequent in the CBA group compared to the RFA group.

Of note, the use of Class III antiarrhythmic drugs—predominantly amiodarone—was comparable between both groups at follow-up, suggesting that AAD therapy is unlikely to have biased the observed recurrence rates.

Overall, both RFA and CBA appear to be highly effective, safe treatment methods with comparable outcomes.

HF frequently coexists with AF, with evidence suggesting a bidirectional relationship where AF predisposes to heart failure, and vice versa. The central pathophysiology involves the development of atrial fibrosis and neurohumoral activation [4]. On one hand, AF may lead to a reduction in diastolic filling times, resulting in decreased cardiac output, functional mitral annular enlargement leading to mitral regurgitation, and an increase in the typical neurohormonal vasoconstrictor excess through elevated plasma endothelin and epinephrine [[16], [17], [18]]. Additionally, AF may contribute to tachycardia-related cardiomyopathy. On the other hand, heart failure induces structural, ultrastructural, and neuroendocrine processes that promote atrial fibrosis due to heightened atrial stress and a vasoconstrictive humoral milieu [19,20]. Consequently, these changes are not only pro-fibrillatory but also cumulative and progressive [4].

It has been approximated, that individuals with HFrEF, who are undergoing both cardiac resynchronization therapy (CRT) and implantable cardioverter-defibrillators (ICD), may experience significant adverse outcomes due to AF. These consequences include ineffective biventricular pacing and insufficient ICD interventions [21]. The EAST-AF net 4 trial recently showed that early rhythm control lowers the risk of adverse events [11]. In addition to pharmacological therapy, maintaining a stable sinus rhythm is a crucial therapeutic strategy for reducing mortality in patients with AF and reduced LVEF today.

Both CBA and RFA qualify equally effective for an interventional ablation approach. Nevertheless, procedural differences were observed. For once, CBA was associated with a significantly shorter procedure time while presenting longer fluoroscopy times and higher dose area products compared to RFA. These observations align with the results of the current trial, confirming previously identified findings [22,23]. Kuck et al. demonstrated a significantly shorter procedure time in CBA compared to RFA (124 vs. 141 min, p < 0.001), along with longer fluoroscopy time (17 vs. 22 min, p < 0.001) [22].

The shorter procedural time in CBA may primarily be attributed to the single-shot design of the CB catheter, which enables en-bloc PVI, compared to the point-by-point lesions produced by RFA. This reduction in procedure and LA time may be particularly relevant in patients with impaired LVEF, as it could help minimize volume overload, sedation duration, and hemodynamic stress during the intervention.

The use of contrast-dye angiographies to verify pulmonary vein occlusion, along with the fluoroscopic guidance of catheters in CBA procedures, contributed to lengthier fluoroscopy times and higher dose-area product values. In contrast, radiofrequency ablation obviates the need for angiography to confirm catheter positioning, and catheter navigation is accomplished through 3D electroanatomical mapping.

In the present study, the overall complication rate in patients with impaired LVEF undergoing PVI via CBA or RFA was comparable to that observed in a non-heart failure population [1]. However, MACCE rates were found to be lower in this cohort compared to the existing literature [12].

This discrepancy could be due to the comparatively healthier and younger subjects in the current study, who had an average CHA_2_DS_2_-VASc-score of 2.6 and a median LVEF of 42 %.

Although the total MACCE rates exhibited no statistically significant difference between the CBA and RFA groups, our investigation identified a lower overall complication rate in the CBA group. This finding is in line with data from Friedmann et al., who reported fewer complications with CBA compared to RFA for de novo procedures in a large, real-world registry population [24]. This variation may be explained by the markedly higher incidence of inguinal hematomas in the RFA group compared to CBA. This trend has been alluded to in previous trials such as FIRE and ICE; however, it has not achieved statistical significance. The higher incidence of groin injuries in RFA procedures, which typically use a smaller maximum sheath diameter (8.5F), compared to those using a larger sheath with a 15F outer diameter, may be linked to the use of a two-sheath system with a radiofrequency catheter and a separate circular mapping catheter in LA [22,25]. This circumstance is concomitant with the utilization of a greater quantity of both total and LA catheters in the RFA group, as evidenced in the current trial. Groin complications can be reduced by ultrasonic guidance [26]. Unfortunately, the dataset does not give evidence if ultrasonic guidance of venous puncture was used more frequently in the CBA group. In the CBA group more intracardiac ultrasound was used to guide the TSP and balloon positioning. The higher use rate of intracardiac ultrasound in this study may suggest that the ultrasound machine was more readily available in the CBA lab. This could potentially explain the lower incidence of groin complications observed in this group, although no systematic data were collected on this aspect.

Although, the majority of patients (82 %) were treated with a “PVI only” approach in this study, it is important to note that there were significant differences in applied ablations beyond PVI between the groups. In the RFA group a significantly higher number of linear lesions were created as compared to the CBA group. This discrepancy could be due to the higher proportion of persistent AF patients in the RFA group, as well as the availability of 3D electroanatomical mapping systems, which facilitate individualized substrate-based ablation strategies. Furthermore, the RFA approach enables targeting of extra-PV triggers and atypical atrial flutters during the same procedure in addition to PVI.

Previous studies, involving both HF and non-HF patients undergoing either CBA or RFA, have reported all-cause rehospitalization rates of up to 40 % [22,27]. However, limited data exist on the rehospitalization rates of patients with reduced LVEF following AF ablation. When comparing the all-cause rehospitalization rates of the present trial to the current literature, which includes both non-HF patients and HF patients, an increase in the all-cause rehospitalization rate can be demonstrated, especially in the RFA population [28]. However, there is no relevant difference regarding re-ablation rates. The rationale for this distinction may be attributed to the increased risk for rehospitalization due to cardiovascular causes besides AF and a more complex patient population.

Regarding the comparison between rehospitalization rates in the CBA and RFA groups, it was suggested that CBA showed potential benefits in decreasing cardiovascular rehospitalization and repeat ablations compared to RFA [22]. In the present trial, no significant difference was found regarding repeat ablations or cardiovascular rehospitalizations. However, the total all-cause rehospitalization rate was significantly lower in the CBA group compared to the RFA group (34.9 % vs. 52.1 %, p < 0.01). This might be attributed to the healthier CBA group, characterized by a lower percentage of severe or disabling EHRA symptom scores, along with a reduced prevalence of valve disease ≥ grade 2 and a lower CHA_2_DS_2_-VASc-Score.

Managing patients with AF and concurrent systolic dysfunction presents notable challenges due to the bidirectional pathophysiologic relationship. Consequently, it is imperative to assess predictors of arrhythmia recurrence in this patient cohort. Numerous factors influencing AF recurrence, such as LA size, age, AF duration, and early AF recurrence, have been delineated in prior studies [1], yet most of these parameters have been derived from investigations encompassing both patients without HF and those with HF. As a result, the scope of prediction analysis specific to patients with reduced LVEF is restricted. Beyond functional parameters, recent work by Long et al. emphasized the relevance of heart failure etiology for outcomes after AF ablation. In a cohort of patients with LVEF < 50 %, those with tachycardia-induced cardiomyopathy showed significantly better rhythm control, lower mortality, and more pronounced improvement in LVEF compared to ischemic and dilated cardiomyopathy [29]. These findings underscore the importance of etiological stratification in interpreting ablation outcomes. In a regression analysis involving 206 HFrEF patients undergoing CBA and RFA, Prahbu et al. demonstrated that ischemic cardiomyopathy serves as an independent parameter [30]. Nonetheless, in the current trial, female sex emerged as the sole independent risk factor for arrhythmia recurrence following AF ablation in multivariate regression analysis. This finding, frequently documented in the AF ablation literature, may be ascribed to various factors. Typically, female AF patients with HF are older and possess more comorbidities than their male counterparts when undergoing catheter ablation [31], consistent with the present trial. This demographic profile may contribute to a heightened burden of atrial fibrosis, thereby facilitating AF [32]. Additionally, postmenopausal women exhibit higher levels of epicardial adipose tissue than matched men, correlating independently with decreased LA voltage and impaired LA transport function [33]. Overall, further research in this domain is warranted and may eventually lead to the development of personalized or sex-tailored ablation strategies.

Ultimately, it is important to note that, despite impaired left ventricular function, patient satisfaction was similarly high in both groups. A total of 65.5 % of patients rated the procedure as either 'overall successful' or 'partially successful', indicating that the majority perceived a subjective benefit from the ablation. This suggests that the presence of left ventricular dysfunction does not significantly impact patient-perceived outcomes and that both major ablation strategies are equally effective in maintaining high levels of patient satisfaction in these patients.

## 5 Limitations

Due to the study design, it is necessary to address some significant limitations. This study constitutes a sub-analysis of the prospective FREEZE cluster-cohort study. While the comparison between CBA and RFA is reassuring for those contemplating such an approach and may contribute insights to the design of prospective clinical studies, it is not a substitute for randomized trials, and additional data is still desirable. Importantly, the lack of EF measurement and information on cardiac biomarkers during follow-up is a notable limitation that could have substantially enhanced the present trial. Ablation strategies beyond PVI differed between groups, with more frequent additional linear lesions in the RFA cohort. This procedural variability may have also influenced outcomes and limits the direct comparability of the two approaches. The study mainly evaluated patient-reported outcomes. Hence, data on asymptomatic AF episodes are incomplete, since Holter-ECG or continuous monitoring were not mandatory and only 79.2 % of patients underwent Holter-ECG during follow-up. No continuous rhythm monitoring with implantable loop recording systems were available. The reader should consider the potential for an overestimation of the clinical success rates in both groups. The initiation of optimal medical treatment or other interventions might have influenced rehospitalization rates as well. Furthermore, arrhythmia recurrences were not differentiated between AF and atrial tachycardia, limiting more detailed analysis regarding the type of recurrence. This study was conducted before the introduction of Angiotensin Receptor-Neprilysin Inhibitors and Sodium-Glucose Cotransporter 2 Inhibitors in heart failure medical therapy. Even though participation of centers was restricted to those with ≥ 50 procedures of either RFA or CBA performed prior the study initiation, research has demonstrated that outcomes are associated with the volume of the center and operators, potentially affecting the results. Notably, almost 15 % of patients in the CBA group were treated with first-generation CB, known to be less effective and no longer available. Since recruitment began before the availability of contact force-sensing catheters, and the availability of advanced RFA catheters was influenced by a safety notice and voluntary field removal by manufacturers in the European Union in the years 2013/2014, further analysis of outcomes based on individual catheter types was precluded.

## 6 Conclusion

In conclusion, both CBA and RFA are effective and safe treatment strategies for AF patients with impaired LVEF. CBA is associated with shorter, less complex procedures and a lower rate of total complications, although it entails higher fluoroscopy times and dose area products compared to RFA. Acute PVI rates are similar, and no significant differences in atrial arrhythmia recurrence are observed during follow-up. Notably, all-cause rehospitalizations are more frequent following RFA. Female sex was identified as the sole independent predictor of arrhythmia recurrence. Consequently, individualized sex-based ablation strategies may be of interest and should be further investigated in larger, prospective cohorts.

The present findings reassure ongoing ablation practices in HF patients with AF and propose further evaluation of the best ablation strategy in this specific AF patient subgroup.

## Ethics approval statement

The local ethics review committees of all centres approved the study. The study complies with the Declaration of Helsinki, and data were collected prospectively. The ethics review committees that approved the study were the following: Ethik-Kommission −Med. Fak. Bonn, Rheinische Friedrich-Wilhelms-Universität, Bonn, Germany; Ethik-Kommission bei der Medizinischen Fakultät, Julius-Maximilians-Universität Würzburg, Würzburg, Germany; Ethik-Kommission, Albert-Ludwigs-Universität Freiburg, Freiburg, Germany; Ethik-Kommission der Ärztekammer Westfalen-Lippe und der Medizinischen Fakultät der Westfälischen Wilhelms Universität, Münster, Germany; Sächsiche Landesärztekammer, Dresden, Germany; Ärztekammer Nordrhein, Düsseldorf, Germany; Ethik-Kommission der Ärztekammer Hamburg, Hamburg, Germany; Ethikkommission der Med. Fak. Heidelberg, Heidelberg, Germany; Ethik-Kommission der Landesärztekammer Rheinland-Pfalz, Mainz, Germany; Ethik-Kommission der Bayerischen Landesärztekammer, München, Germany; Landesärztekammer Baden-Württemberg, Stuttgart, Stuttgart, Germany; Ethik-Kommission OLVG, Amsterdam, Netherlands; Ethik-Kommission Arrixaca, El Palmar (Murcia), Spain; Pharma-ethics, Lyttelton, Zuid Africa; Ethikkommission des Landes Oberösterreich, Linz, Austria.

**Patient consent statement**

Informed consent was obtained from all patients in the FREEZE Cohort study.

**Clinical trial registration**

Clinical trial registration NCT01360008 (first registration 25/05/2011).

**Funding**

The FREEZE Cohort Trial is an investigator-initiated study; the non-profit foundation ‘Stiftung Institut fuer Herzinfarktforschung’ institute (Ludwigshafen, Germany) conducted the trial and was reimbursed by Medtronic GmbH, Meerbusch, Germany for their services. The participants, investigators, centers or members of the steering committee received no compensation.

**Data availability statement:** The data that support the findings of this study are available from the corresponding author upon reasonable request.

## CRediT authorship contribution statement

**J. Pongratz:** Writing – review & editing, Writing – original draft, Investigation, Formal analysis. **K.-H. Kuck:** Writing – review & editing, Investigation. **A. Metzner:** Writing – review & editing, Investigation. **U. Hink:** Writing – review & editing, Investigation. **A. Garcia Alberola:** Writing – review & editing, Investigation. **R. Borchard:** Writing – review & editing, Investigation. **G. Nölker:** Writing – review & editing, Investigation. **M. Kuniss:** Writing – review & editing, Investigation. **R.R. Tilz:** Writing – review & editing, Investigation. **J.W. Schrickel:** Writing – review & editing, Investigation. **A. Thornton:** Writing – review & editing, Investigation. **D. Thomas:** Writing – review & editing, Investigation. **H.A. Katus:** Writing – review & editing, Investigation. **R. Zahn:** Writing – review & editing, Investigation. **S. Spitzer:** Writing – review & editing, Investigation. **J.J. Souza:** Writing – review & editing, Investigation. **J. Brachmann:** Writing – review & editing, Investigation. **J. Tebbenjohanns:** Writing – review & editing, Investigation. **L.-Q. Wu:** Writing – review & editing, Investigation. **W.P. Obel:** Writing – review & editing, Investigation. **G. Groschup:** Writing – review & editing, Investigation. **C. Stellbrink:** Writing – review & editing, Investigation. **K.R.J. Chun:** Writing – review & editing, Investigation. **J.-H. Gerds-Li:** Writing – review & editing, Investigation. **A. Stanley:** Writing – review & editing, Investigation. **R.R. Gopal:** Writing – review & editing, Investigation. **L. Lickfett:** Writing – review & editing, Investigation. **A. Lubinski:** Writing – review & editing, Investigation. **B. Schumacher:** Writing – review & editing, Investigation. **C. Steinwender:** Writing – review & editing, Investigation. **H.A. Franke:** Writing – review & editing, Investigation. **T. Ouarrak:** Writing – review & editing, Methodology, Investigation, Formal analysis. **U. Dorwarth:** Writing – review & editing, Investigation. **J. Senges:** Writing – review & editing, Project administration, Methodology, Conceptualization. **E. Hoffmann:** Writing – review & editing, Investigation. **F. Straube:** Writing – review & editing, Investigation.

## Declaration of competing interest

The authors declare the following financial interests/personal relationships which may be considered as potential competing interests: Dr. Straube received honoraria for lectures from Medtronic, Boston Scientific, Philips, Bristol-Myers-Squibb, and Astra Zeneca outside the submitted work. Dr. Dorwarth reports honoraria for lectures from Medtronic Inc., Bristol-Myers-Squibb, and Astra Zeneca, outside the submitted work. Dr. Hoffmann is head of the department; the department received compensation for participation in clinical research trials outside the submitted work from Abbott, Bayer, Biotronik, Boehringer Ingelheim, Edwards, Elixier, Medtronic, and Stentys; and educational grants from Biotronik, Shockwave. Dr. Thomas reports receiving lecture fees/honoraria from AstraZeneca, Bayer Vital, Boehringer Ingelheim Pharma, Bristol-Myers Squibb, Daiichi Sanyo, Medtronic, Pfizer Pharma, Sanofi-Aventis, St. Jude Medical/Abbott and ZOLL CMS. All other authors had nothing to declare.
