# Supplementary material for: Comparative analysis of cryoballoon versus radiofrequency catheter ablation in atrial fibrillation patients with impaired left ventricular ejection fraction
Source: Int J Cardiol Heart Vasc. 2025 Jun 19;59:101721. doi: 10.1016/j.ijcha.2025.101721 (PMC12221379; doi:10.1016/j.ijcha.2025.101721)
Supplement: Supplementary Methods [file mmc1.docx]

**Supplementary Methods**

**Radiofrequency Ablation**

Radiofrequency ablation procedures adhered to internationally established standards. Pulmonary vein isolation (PVI) was performed using a point-by-point technique. The choice of ablation catheter and 3D mapping system was left to the discretion of the operator, with all CE-approved systems permitted. Additional lesions beyond PVI were allowed as clinically indicated. During the study period, multiple RF catheters from different manufacturers were in use. Although contact-force sensing catheters became available over time, their use was not consistent across centers, and documentation within the electronic case report form was incomplete regarding catheter type and mapping system.

**Cryoballoon Ablation**

Cryoballoon ablation was performed using an over-the-wire technique, in line with current standards of care. Three generations of cryoballoons (Arctic Front™, Advance™, and Advance ST™, Medtronic Inc.) were used depending on availability. The primary goal was complete PVI, with additional cryothermal or RF touch-up lesions permitted when necessary. Phrenic nerve monitoring during ablation of right-sided PVs was mandatory, achieved through pacing or fluoroscopy. A circular mapping catheter (Achieve™, 15 or 20 mm) was used to assess PV potentials and guide balloon positioning. The 28-mm balloon was preferred; use of the 23-mm balloon required prior imaging (cardiac computed tomography, cardiac magnetic resonance imaging, or PV angiography) to evaluate anatomy, and was performed at the discretion of the participating centers.
