# Supplementary material for: Comparative analysis of cryoballoon versus radiofrequency catheter ablation in atrial fibrillation patients with impaired left ventricular ejection fraction
Source: Int J Cardiol Heart Vasc. 2025 Jun 19;59:101721. doi: 10.1016/j.ijcha.2025.101721 (PMC12221379; doi:10.1016/j.ijcha.2025.101721)
Supplement: Supplementary Table 1 [file mmc2.docx]

**Supplement table 1: Adverse events until hospital discharge**

|  | **Total (n=256)** | **Group A (n=118)** | **Group B (n=138)** | **p-value** |
| --- | --- | --- | --- | --- |
| **Procedural complications (%)** | 24/254 (9.4) | 6/117 (5.1) | 18/137 (13.1) | **< 0.05** |
| **Hospital MACCE** | 1/255 (0.4) | 0/118 (0.0) | 1/137 (0.7) | 0.35 |
| **Death (%)** | 0/255 (0.0) | 0/118 (0.0) | 0/137 (0.0) | - |
| **Myocardial infarction (%)** | 0/255 (0.0) | 0/118 (0.0) | 0/137 (0.0) | - |
| **Stroke (%)** | 1/255 (0.4) | 0/118 (0.0) | 1/137 (0.7) | 0.35 |
| **Major complications (%)** | 5/253 (2.0) | 1/116 (0.9) | 4/137 (2.9) | 0.24 |
| **Persistent phrenic nerve palsy (%)** | 0/253 (0.0) | 0/116 (0.0) | 0/137 (0.0) | - |
| **Tamponade or pericardial effusion treated by drainage or surgery (%)** | 2/253 (0.8) | 1/116 (0.9) | 1/137 (0.7) | 0.92 |
| **Resuscitation (%)** | 0/253 (0.0) | 0/116 (0.0) | 0/137 (0.0) | - |
| **TIA < 24h (%)** | 0/253 (0.0) | 0/116 (0.0) | 0/137 (0.0) | - |
| **Pulmonary embolism (%)** | 0/253 (0.0) | 0/116 (0.0) | 0/137 (0.0) | - |
| **Hemato/pneumothorax (%)** | 0/253 (0.0) | 0/116 (0.0) | 0/137 (0.0) | - |
| **Groin complications treated interventionally or with surgery (%)** | 3/253 (1.2) | 0/116 (0.0) | 3/137 (2.2) | 0.11 |
| **Minor complications (%)** | 19/254 (7.5) | 5/117 (4.3) | 14/137 (10.2) | 0.07 |
| **Transient phrenic nerve palsy (%)** | 1/255 (0.4) | 1/118 (0.8) | 0/137 (0.0) | 0.28 |
| **Pericardial effusion or pericarditis (%)** | 1/255 (0.4) | 0/118 (0.0) | 1/137 (0.7) | 0.35 |
| **Groin complications treated conservatively (%)** | 8/254 (3.1) | 2/117 (1.7) | 6/137 (4.4) | 0.22 |
| **Inguinal hematoma treated conservatively (%)** | 6/254 (2.4) | 0/117 (0.0) | 6/137 (4.4) | **<0.05** |
| **AV fistula or spurious aneurysm treated conservatively (%)** | 3/255 (1.2) | 2/118 (1.7) | 1/137 (0.7) | 0.48 |
| **Other problems at puncture side treated conservatively (%)** | 1/255 (0.4) | 1/118 (0.8) | 0/137 (0.0) | 0.28 |
| **Cardiac complication (arrhythmia, decompensation) (%)** | 2/255 (0.8) | 1/118 (0.8) | 1/137 (0.7) | 0.92 |
| **Systemic infection (%)** | 1/255 (0.4) | 0/118 (0.0) | 1/137 (0.7) | 0.35 |
| **Esophageal lesion (%)** | 1/255 (0.4) | 0/118 (0.0) | 1/137 (0.7) | 0.35 |
| **Other complications (%)** | 5/255 (2.0) | 1/118 (0.8) | 4/137 (2.9) | 0.23 |

n (%), Mean ± SD, or Median (Quartiles) as appropriate according to the test of normal distribution. MACCE: major adverse cardiac and cerebrovascular events, TIA: transient ischemic attack.
