# Supplementary material for: Comparative analysis of cryoballoon versus radiofrequency catheter ablation in atrial fibrillation patients with impaired left ventricular ejection fraction
Source: Int J Cardiol Heart Vasc. 2025 Jun 19;59:101721. doi: 10.1016/j.ijcha.2025.101721 (PMC12221379; doi:10.1016/j.ijcha.2025.101721)
Supplement: Supplementary Table 2 [file mmc3.docx]

**Supplement table 2: Adverse events during follow-up**

|  | **Total (n=256)** | **Group A (n=118)** | **Group B (n=138)** | **p-value** |
| --- | --- | --- | --- | --- |
| **Posthospital total complications (%)** | 25/185 (13.5) | 10/87 (11.5) | 15/98 (15.3) | 0.45 |
| **Posthospital MACCE** | 11/185 (5.9) | 3/87 (3.4) | 8/98 (8.2) | 0.18 |
| **Death (%)** | 6/244 (2.5) | 1/110 (0.9) | 5/134 (3.7) | 0.16 |
| **Causes of death** |  |  |  | 0.34 |
| **Cardiovascular (%)** | 2/6 (33.3) | 1/1 (100.0) | 1/5 (20.0) |  |
| **Non-Cardiovascular (%)** | 1/6 (16.7) | 0/1 (0.0) | 1/5 (20.0) |  |
| **Unknown (%)** | 3/6 (50.0) | 0/1 (0.0) | 3/5 (60.0) |  |
| **Posthospital myocardial infarction (%)** | 0/178 (0.0) | 0/86 (0.0) | 0/92 (0.0) | - |
| **Posthospital Stroke (%)** | 5/180 (2.8) | 2/86 (2.3) | 3/94 (3.2) | 0.72 |
| **Posthospital major complications (%)** | 5/177 (2.8) | 1/85 (1.2) | 4/92 (4.3) | 0.20 |
| **TIA (%)** | 0/179 (0.0) | 0/85 (0.0) | 0/94 (0.0) | - |
| **PV Stenosis (%)** | 3/181 (1.7) | 0/86 (0.9) | 3/95 (3.2) | 0.10 |
| **Atrio-esophageal fistula (%)** | 0/180 (0.0) | 0/85 (0.0) | 0/95 (0.0) | - |
| **Persistent phrenic nerve palsy (%)** | 0/181 (0.0) | 0/86 (0.0) | 0/95 (0.0) | - |
| **Severe bleeding (%)** | 2/179 (1.1) | 1/86 (1.2) | 1/93 (1.1) | 0.96 |
| **Pericardial effusion treated interventionally or with surgery (%)** | 0/179 (0.0) | 0/85 (0.0) | 0/94 (0.0) | - |
| **Pulmonary embolism (%)** | 0/178 (0.0) | 0/86 (0.0) | 0/92 (0.0) | - |
| **Posthospital minor complications (%)** | 14/177 (7.9) | 7/85 (8.2) | 7/92 (7.6) | 0.88 |
| **Moderate bleeding (%)** | 2/178 (1.1) | 1/85 (1.2) | 1/93 (1.1) | 0.95 |
| **Pericardial effusion treated conservatively (%)** | 1/179 (0.6) | 1/85 (1.2) | 0/94 (0.0) | 0.29 |
| **Syncopes (%)** | 3/179 (1.7) | 2/87 (2.3) | 1/92 (1.1) | 0.53 |
| **Phlebothrombosis (%)** | 1/178 (0.6) | 0/86 (0.0) | 1/92 (1.1) | 0.33 |
| **Inguinal problems (%)** | 8/178 (4.5) | 3/84 (3.6) | 5/94 (5.3) | 0.57 |

n (%), Mean ± SD, or Median (Quartiles) as appropriate according to the test of normal distribution. MACCE: major adverse cardiac and cerebrovascular events, TIA: transient ischemic attack.
