# Supplementary material for: Comparative analysis of cryoballoon versus radiofrequency catheter ablation in atrial fibrillation patients with impaired left ventricular ejection fraction
Source: Int J Cardiol Heart Vasc. 2025 Jun 19;59:101721. doi: 10.1016/j.ijcha.2025.101721 (PMC12221379; doi:10.1016/j.ijcha.2025.101721)
Supplement: Supplementary Table 3 [file mmc4.docx]

**Supplement table 3: Predictors of atrial arrhythmia recurrence**

|  | **OR** | **95% CI** | **p-value** |
| --- | --- | --- | --- |
| **RFA** | 1.30 | 0.650-2.606 | 0.46 |
| **LVEF, %** | 0.96 | 0.912-1.009 | 0.11 |
| **Female sex** | 2.53 | 1.147-5.592 | **0.02** |
| **Age, years** | 1.00 | 0.967-1.043 | 0.82 |
| **BMI > 35 kg/m^2^** | 0.37 | 0.092-1.478 | 0.16 |
| **LA diameter, mm*** | 1.01 | 0.959-1.067 | 0.67 |
| **Paroxysmal AF** | 0.64 | 0.313-1.294 | 0.21 |

This table lists all the parameters included in the logistic regression analysis for calculating predictors of atrial arrhythmia recurrence. The model identified female sex as the sole independent risk factor for atrial arrhythmia recurrence, with a p-value of 0.02.

RFA: radiofrequency ablation, LVEF: left atrial ejection fraction, BMI: body mass index, LA: left atrial.

*Determined via transthoracic echocardiography.
